# The Jordan Stillbirth and Neonatal Mortality Surveillance (JSANDS) System: Evaluation Study

**DOI:** 10.2196/29143

**Published:** 2021-07-21

**Authors:** Yousef Khader, Mohammad Alyahya, Ziad El-Khatib, Anwar Batieha, Nihaya Al-Sheyab, Khulood Shattnawi

**Affiliations:** 1 Department of Public Health Faculty of Medicine Jordan University of Science and Technology Irbid Jordan; 2 Karolinska Institutet Stockholm Sweden

**Keywords:** stillbirths, neonatal deaths, surveillance, eHealth, electronic health data, electronic surveillance, pediatrics, maternity, mortality, mortality surveillance, Ministry of Health, health data

## Abstract

**Background:**

The Jordan Stillbirth and Neonatal Mortality Surveillance (JSANDS) is an electronic surveillance system that automatically transfers the data on births, stillbirths, and neonatal deaths to the concerned authorities in the Ministry of Health. JSANDS was implemented and tested in 5 maternity hospitals during the period spanning May 2019 through December 2020.

**Objective:**

This study aimed to evaluate the usefulness and performance of JSANDS to register births, stillbirths, and neonatal deaths, and determine their causes. Specifically, this study examined the JSANDS attributes of acceptability, simplicity, flexibility, stability, representativeness, sustainability, penetration, data quality, sensitivity, and adoption.

**Methods:**

An evaluation study was conducted after 18 months of the JSANDS implementation using the Updated Guidelines for Evaluating Public Health Surveillance Systems. The evaluation focused on how well the system operated to meet its purpose and objectives. The indicators assessing the system attributes were scored on a Likert scale. Each indicator and overall attribute percentage score was represented as score rank and interpreted as excellent (score ≥80%), good (score ≥60 and <80%), average (score ≥40 and <60%), and poor (score <40%).

**Results:**

A total of 270 health care professionals participated in this study and evaluated the system performance. The system users rated the usefulness of JSANDS as excellent (percentage score=85.6%). The overall acceptability (percentage score=82.3%), flexibility (percentage score=80.2%), stability (percentage score=80.0%), and representativeness (percentage score=86.6%) were also rated excellent. The overall simplicity was scored good (percentage score=75.4%). All participants were trained on JSANDS and used it in the past 12 months. Of the 270 respondents, 219 (86.2%) reported that they intend to continue using the JSANDS system to register neonatal deaths and stillbirths in the future. All variables in JSANDS had complete data with no missing values.

**Conclusions:**

The performance of JSANDS in registering all stillbirths and neonatal deaths as well as their causes was excellent. Almost all attributes and indicators of JSANDS functionality were rated excellent. JSANDS can be scaled up to cover all maternity hospitals in Jordan. The potential for scaling up the system is very high for many reasons, including its usefulness, simplified stillbirth and neonatal death review tools, and ease of the reporting process.

## Introduction

### Maternal and Neonatal Services in Jordan

In Jordan, maternal and neonatal services are provided by the public sector (Ministry of Health and Royal Medical Services), private sector, and teaching hospitals. A total of 27 Ministry of Health hospitals provide birth services, with nearby maternal and child health centers providing antenatal and postnatal care. A recent study in Jordan [1] showed that the quality of maternal and newborn care in many Jordanian hospitals is relatively acceptable. Nonetheless, some maternity hospitals have some deficiencies, including shortages of skilled and competent birth attendants; lack of pivotal protocols, policies, and guidelines necessary for optimal care; lack of optimal thorough antenatal care; and lack of necessary supplies, drugs, equipment, and resources during labor and early postnatal care. Moreover, the study reported that some health care professionals are not regularly trained on routine and special care needed for labor and the early postpartum period in both normal and complicated births.

### Jordan Stillbirth and Neonatal Mortality Surveillance

A well-functioning perinatal and neonatal (PNN) mortality surveillance supports the delivery of health services by ensuring the production, analysis, dissemination, and use of reliable and timely information on PNN mortality determinants and quality of care [2-5]. Therefore, a comprehensive electronic surveillance system to register and accurately report stillbirths and neonatal deaths and their causes was developed and implemented.

The Jordan Stillbirth and Neonatal Mortality Surveillance (JSANDS) is an electronic surveillance system that automatically transfers the data on births, stillbirths, and neonatal deaths to the concerned authorities in the Ministry of Health. The development of the surveillance system was informed by the findings of stakeholder analysis and formative qualitative and quantitative research among health professionals, local communities, families, and women [4-7]. The main objectives of this system are to measure the burden of PNN mortality, identify their causes, assess trends in these deaths, and identify risk groups. The data collected by JSANDS are useful in guiding the planning, implementation, and evaluation of programs aiming at prevention and reducing PNN deaths; in prioritizing the allocation of health resources; in formulating research hypotheses; and in providing a basis for epidemiologic research. The JSANDS dashboard displays useful information on mortality indicators, including fetal death rate, neonatal mortality rate, perinatal death rate, and causes of stillbirths and neonatal deaths. These indicators are calculated as an overall score and stratified by social and clinical variables. The potential users of the data include policy makers and decision makers at the Ministry of Health, clinicians, researchers, and other health care providers.

### Essential Data Reported by JSANDS

JSANDS collects data on demographic, clinical, maternal, and other relevant characteristics for each live born baby, stillbirth, and neonatal death to allow detailed exploration of the causes of perinatal mortality in Jordan, to calculate and report indicators, to make comparisons between organizations, and to provide a clearer insight into the risk factors most commonly associated with stillbirth or neonatal death. In JSANDS, stillbirth is defined as a baby delivered at or after 24 weeks of gestation or having a birthweight of ≥500 grams and showing no signs of life, irrespective of when the death occurred. Stillbirths include antepartum stillbirth (death before the onset of labor) and intrapartum stillbirth (known to be alive at the onset of labor). Neonatal death is defined as a live born infant at ≥ 24 weeks of gestation or with a birthweight of ≥ 500 grams who died before 28 complete days after birth. Perinatal death is defined as fetal death after 24^+0^ completed weeks of gestation and before 7 complete days after birth.

### The Procedure of Death Notification and Assigning Cause of Death

JSANDS reports all live births, stillbirths, and neonatal deaths occurring in each hospital. The obstetrician attending the delivery and the midwife are responsible for entering the necessary data for deliveries and live births. The clinician conducting the delivery of a stillbirth has the primary responsibility to fill the form for the stillbirth, assign the cause of death, and write the International Classification of Diseases Tenth Revision (ICD-10) code accordingly. For neonatal deaths, the clinician and the nurse attending the neonatal deaths are responsible for completing the needed information on each death, including cause of death according to the ICD-10. JSANDS uses the ICD-10 codes to provide a common language for reporting and monitoring diseases [8]. The causes of deaths are set out as follows: main disease or condition in the fetus or infant, for which the single most important and main disease or condition of the infant or fetus who has died is entered; other diseases or conditions in the fetus or infant; main maternal disease or condition affecting the fetus or infant; or other maternal diseases or conditions affecting the fetus or infant.

### Objectives of the Evaluation

This study aimed to evaluate the usefulness and performance of JSANDS to register births, stillbirths, and neonatal deaths, and to determine their causes. Specifically, this study examined the acceptability, simplicity, flexibility, stability, representativeness, sustainability, penetration, data quality, sensitivity, and adoption of the system.

## Methods

### Settings

JSANDS was implemented and tested in 5 maternity hospitals during the period spanning May 2019 through December 2020. These 5 selected hospitals from 3 major governorates cover the vast majority of births and deaths that occur in all regions in the north, east, and south of Jordan. In particular, we included one university teaching, referral hospital in Northern Jordan, which receives labor and delivery cases from all regions and suburbs in Northern Jordan. We also included a governmental hospital affiliated with the Ministry of Health in Northern Jordan, which is specialized for maternal-related issues, including births, and also receives maternal deliveries from all sectors in the region. A large, private hospital in the north of Jordan was also included from several private and governmental sectors in the region. In the northeast region, we included a large specialized hospital for maternal and child health that covers the whole Al-Mafraq governorate. Finally, we selected another specialized referral hospital in the southern region of Jordan which covers the majority of births within the region.

### Study Design

An evaluation study was conducted to ensure that PNN deaths, their causes, and their contributing factors, including social determinates, were being reported and monitored efficiently and effectively by JSANDS after 18 months of the implementation. The evaluation focused on how well the system operated to meet its purpose and objectives. We used evaluation guidelines of the Updated Guidelines for Evaluating Public Health Surveillance Systems developed by the Centers for Disease Control and Prevention (CDC) [9]. The evaluation involved an assessment of the usefulness of the system and other system attributes including acceptability, simplicity, flexibility, stability, representativeness, sustainability, penetration, data quality, sensitivity, and adoption “uptake”. These attributes were of concern for the hardware and software, standard user interface, standard data format and coding, appropriate quality checks, and adherence to confidentiality and security standards. For the purpose of evaluation, we engaged all JSANDS users in this study including midwives, nurses, and physicians. The users included those who enter the data, assign causes of death, and use the system for monitoring the deaths in the selected hospitals.

A structured self-reported questionnaire was developed and included specific indicators to assess the system attributes. The indicators were developed based on the literature review of previous public health surveillance evaluations. The questions were developed by 3 experts who have experience in the development and evaluation of surveillance systems, and the content validity was established by a group of 5 specialists in neonatology, obstetrics, and epidemiology. The questionnaire was pilot tested on 12 JSANDS users whose responses were not included in the main study. Minimal changes, mostly related to clarity of questions, were made on these questions. The number of used indicators varied according to the attribute measured. Participants were asked to rate the degree to which they agreed with the attributes’ specific indicators using a 5-point Likert scale (1=strongly disagree, 2=disagree, 3=neutral, 4=agree, and 5=strongly agree), with a higher score indicating a better performance on the studied attribute.

### Operation Definitions of the System Attributes

#### Level of Usefulness

The system’s users were requested to rate 9 indicators of usefulness using a 5-point Likert scale. The system was considered useful if it was able to provide estimates of the PNN mortality rate, the causes of deaths, and the social determinants of deaths; report equity indicators; facilitate assessment of the effect of prevention and control programs; improve the delivery of maternal and child services; and stimulate research.

#### Acceptability

Seven indicators of acceptability were rated by users. The indicators assessed the user’s willingness to use the JSANDS system with and without incentives, their intention to continue using JSANDS in the future, and their satisfaction with the system.

#### Simplicity

The simplicity of the system included both the structure and ease of operation. Eight simplicity indicators were assessed by users. The indicators assessed the ease of use of the data entry interface, comprehensibility of the variables, comprehensibility of the case definitions, the need for frequent training, and the ease in obtaining the data.

#### Flexibility

Flexibility referred to whether the system can accommodate new health-related events and changes in case definitions and whether it can be easily integrated with other systems. Ten indicators were assessed by users. The indicators referred the possibility of adding new variables with minimum cost and effort, the functionality of the system after funding withdrawal, the ability of the restructuring the system to monitor maternal deaths, the ability of the restructuring the system to monitor congenital anomalies, the ability of the restructuring the system to monitor infant deaths, the possibility of integrating JSANDS with other systems, the ability of the system to accommodate changes in the case definitions, the ability of the system to accommodate ICD revision, the functionality of the system if used in other countries, and the functionality of the system if used in other health sectors.

#### Stability

Stability was considered to be the ability of the system to properly collect, manage, and provide data without failure. Five indicators were used to assess the stability. The indicators assessed the stability of the system after withdrawal of sponsors’ support, availability of resources for maintenance, and availability of trained staff.

#### Representativeness

Five indicators were used to assess the usability of the system in other hospitals and the performance of the system to register all births, stillbirths, and neonatal deaths that occurred in the selected hospitals during the study period.

#### Sustainability

Sustainability referred to the extent to which the system could be maintained or institutionalized within the implementing hospitals. Sustainability was assessed as the success of the research team’s activities and the attempt to sustain the system and scale it up to other health facilities, as well as leadership support for sustainability.

#### Adoption “Uptake”

Adoption uptake was considered to be the intention to continue using the system with or without incentives as assessed by the responses of users engaging with the system at the time of the survey.

#### Penetration

Penetration was defined as the integration of the surveillance system within hospitals. It was evaluated by calculating the proportion of health professionals who used the surveillance system to report births and PNN deaths relative to the number of trained health professionals.

#### Data Quality

The database of all registered births and deaths during the study period was reviewed and checked for data quality. The percentage of the missing values in the core variables was calculated.

The sensitivity and positive predictive value of the surveillance system were not calculated because autopsies are not routinely performed for perinatal deaths in Jordan and because the number of perinatal deaths occurring in the community is not known.

### Adherence to Ethical Standards

Ethical approval for the JSANDS evaluation was obtained from the Institutional Review Board at Jordan University of Science and Technology. A written informed consent was obtained from all JSANDS users who participated in this study. Every effort was made to protect the confidentiality and the identity of participants.

### Data Analysis and Interpretation

The indicators assessing the system attributes were scored on a Likert scale from 1 (“strongly disagree”) to 5 (“strongly agree”). For each indicator, the percentage score was calculated as follows: (sum of all respondents’ scores for each indicator/[maximum score of indicator × number of respondents]) ×100

The overall attribute percentage score was calculated as follows: (sum of all respondents’ scores for all indicators/[number of indicators × maximum score of indicator × number of respondents) ×100. Each indicator and overall attribute percentage score were interpreted as a ranked score as follows: excellent (score ≥80%), good (score ≥60 and <80%), average (score ≥40 and <60%), and poor (score <40%).

## Results

### JSANDS Users’ Characteristics

Of the 270 JSANDS’s users, 254 (94.1%; 13 men and 241 women) health care professionals participated in this study and evaluated the system performance. Their age ranged from 23 to 65 years, with a mean age of 33.2 years (SD 7.0). Among all respondents, 16 (6.3%) were pediatricians, 23 (9.1%) were obstetricians, 126 (49.6%) were pediatric nurses, and 89 (35.0%) were midwives. All participants were trained on the surveillance system.

### Level of Usefulness

The system users rated the usefulness of JSANDS as excellent (percentage score=85.6%). Table 1 shows the ratings of the 9 usefulness indicators. All indicators were rated as excellent. The system provides estimates of the overall PNN mortality rate and the causes of death and those according to other social and clinical determinants. Moreover, the system reports data on other maternal and child health indicators, such as cesarean section rate, preterm delivery rate, and low–birth-weight delivery rate.

**Table 1:** The percentage score and rank of the JSANDS usefulness indicators.

Usefulness indicators	Score, mean (SD)	Percentage score (%)	Rank
The data in the system can be used to estimate the magnitude of stillbirths and neonatal mortality	4.4 (0.7)	87.8	Excellent
The data in the system can be used to monitor the trend of stillbirths and neonatal mortality	4.4 (0.7)	88.3	Excellent
The data in the system can be used to identifying newborns at high risk of dying	4.3 (0.8)	86.1	Excellent
The data can be used for planning the resources for prevention and control neonatal deaths	4.2 (0.8)	85.0	Excellent
The data in the system can be used to update and develop national policy strategy	4.2 (0.8)	83.5	Excellent
The data in the system can be used to assess the impact of any interventions in your hospital	4.3 (0.8)	85.1	Excellent
JSANDS^a^ is able to detect unusual increases in the number of stillbirths and deaths	4.3 (0.7)	86.9	Excellent
JSANDS encourages health workers to prevent neonatal deaths	4.1 (1.0)	82.3	Excellent
JSANDS encourages health workers to improve neonatal health services	4.3 (0.8)	85.7	Excellent
Overall usefulness	4.3 (0.8)	85.6	Excellent

^a^JSANDS: Jordan Stillbirth and Neonatal Mortality Surveillance.

### The System Performance

#### Acceptability

The overall acceptability was scored excellent (percentage score=82.3%). All indicators of the acceptability were rated excellent (Table 2) except for 1 indicator, “Using JSANDS is time-consuming,” which received the lowest percentage score (71.8%) with a “good” rank.

**Table 2:** The percentage score and rank of the JSANDS acceptability indicators.

Acceptability indicators	Score, mean (SD)	Percentage score (%)	Rank
I am very willing to use the JSANDS^a^ system and register neonatal deaths and stillbirths	4.3 (0.9)	86.1	Excellent
I am very willing to use the system and register neonatal deaths and stillbirths without having any incentives	4.2 (1.2)	83.1	Excellent
I will continue using the JSANDS system to register neonatal deaths and stillbirths in future	4.2 (0.9)	83.5	Excellent
I am satisfied with the system	4.1 (0.9)	82.1	Excellent
My colleagues are willing to use JSANDS	4.3 (0.8)	86.2	Excellent
The JSANDS team respond to our suggestions and recommendations	4.1 (0.9)	81.8	Excellent
Using JSANDS is not time-consuming	3.6 (1.5)	71.8	Good
Overall acceptability	4.1 (1.0)	82.3	Excellent

^a^JSANDS: Jordan Stillbirth and Neonatal Mortality Surveillance.

#### Simplicity

The overall simplicity was scored as good (percentage score=75.4%). Of the 8 simplicity indicators, 3 indicators were rated excellent and the remaining 5 indicators were rated good (Table 3). The 2 indicators, “The focal points don’t need continuous and frequent trainings to use the system” and “The JSANDS doesn’t require too much information to register neonatal death and stillbirths,” were rated the lowest.

**Table 3:** The percentage score and rank of the JSANDS acceptability indicators.

Simplicity indicators	Score, mean (SD)	Percentage score (%)	Rank
The JSANDS^a^ data entry interface fields are easy to fill	4.4 (0.9)	87.2	Excellent
The variables in the JSANDS data entry interface are easy to understand	4.3 (0.9)	86.5	Excellent
Data entry in the JSANDS is time-consuming	3.6 (1.5)	71.2	Good
Collecting detailed information of cases don’t require telephone contact or home visit	3.4 (1.5)	68.7	Good
The case definitions (definition of stillbirth and neonatal death) used by JSANDS are easy to understand	4.1 (1.0)	82.1	Excellent
The focal points don’t need continuous and frequent trainings to use the system	3.3 (1.4)	65.9	Good
The sources of information on mortality and causes of deaths are easy to obtain	3.9 (1.3)	77.4	Good
The JSANDS doesn’t require too much information to register neonatal death and stillbirths	3.3 (1.5)	65.5	Good
Overall simplicity	3.8 (1.3)	75.4	Good

^a^JSANDS: Jordan Stillbirth and Neonatal Mortality Surveillance.

#### Flexibility

The ratings of the flexibility indicators are shown in Table 4. Overall, flexibility was scored excellent (percentage score=80.2%) by the system users. Seven out of ten flexibility indicators were rated excellent, and the rest were rated good (Table 4).

**Table 4:** The percentage score and rank of the JSANDS flexibility indicators.

Flexibility indicators	Mean score SD	Percentage score (%)	Rank
New variables can be added to the system with minimum cost and effort	3.7 (1.1)	74.7	Good
The JSANDS^a^ system does not need continuous funding	3.9 (1.4)	77.7	Good
The system can be restructured easily to register maternal deaths	4.0 (0.9)	80.3	Excellent
The system can be restructured to register congenital anomalies	4.2 (0.8)	84.0	Excellent
The system can be restructured easily to register infant mortality (deaths below 1 year)	4.1 (0.9)	82.7	Excellent
The system can be integrated in the Hakeem system or your health information system easily	3.8 (1.2)	75.9	Good
The JSANDS system can accommodate changes in case definition of neonatal deaths	4.1 (0.8)	82.4	Excellent
The JSANDS system can accommodate new ICD^b^ revisions, such as the ICD-11	4.0 (0.8)	80.0	Excellent
The JSANDS system can be used in any country with minimal changes	4.1 (0.9)	81.9	Excellent
The JSANDS system can be used in any hospital in Jordan	4.1 (1.0)	82.8	Excellent
Overall flexibility	4.0 (1.0)	80.2	Excellent

^a^JSANDS: Jordan Stillbirth and Neonatal Mortality Surveillance.

^b^ICD: International Classification of Diseases.

#### Stability

The overall stability score was rated excellent (percentage score=80.0%) (Table 5). Three indicators were rated excellent, and two indicators were rated good. The indicator, “The system is stable even when sponsors withdrawal support” received the lowest score (72.0%).

**Table 5:** The percentage score and rank of the JSANDS stability indicators.

Stability indicators	Score, mean (SD)	Percentage score (%)	Rank
There are planned resources for maintenance of the JSANDS^a^ system	4.1 (1.0)	82.5	Excellent
The system is stable even sponsors withdrawal support	3.6 (1.4)	72.0	Good
There are trained staff in the hospital to use the system	4.5 (0.6)	90.2	Excellent
It is easy to know how to use the system	4.5 (0.7)	90.4	Excellent
JSANDS is not affected by the turnover of trained staff	4.0 (1.4)	79.8	Good
Overall stability	4.1 (1.0)	80.8	Excellent

^a^JSANDS: Jordan Stillbirth and Neonatal Mortality Surveillance.

#### Representativeness

The overall representativeness score was rated excellent (percentage score=86.6%) (Table 6). All representativeness indicators were rated excellent.

**Table 6:** The percentage score and rank of the JSANDS representativeness indicators.

Representativeness indicators	Score, mean (SD)	Percentage score (%)	Rank
The system can be used in any MoH^a^ hospital	4.2 (0.8)	84.8	Excellent
The system can be used in any Royal Medical services hospital	4.3 (0.8)	85.0	Excellent
The JSANDS^b^ system registered all births occurred in the hospital in the past period	4.3 (0.8)	86.6	Excellent
The JSANDS system registered all stillbirths occurred in the hospital in the past period	4.4 (0.7)	88.1	Excellent
The JSANDS system registered all neonatal deaths that occurred in the hospital in the past period	4.4 (0.7)	88.9	Excellent
Overall representativeness	4.3 (0.7)	86.6	Excellent

^a^MoH: Minsitry of Health.

^b^JSANDS: Jordan Stillbirth and Neonatal Mortality Surveillance.

#### Sustainability

The policymakers at the Jordan Ministry of Health and the UNICEF (The United Nations Children's Fund) team were supportive of scaling up JSANDS to other hospitals in Jordan. The Secretory General formed a steering committee for scaling up the system, and a letter signed by the Minister of Health was sent to the other hospitals’ directors to implement JSANDS and use it for registration of neonatal deaths and stillbirths.

#### Adoption Uptake

All participants were trained on JSANDS and used it in the past 12 months. Of the 270 respondents, 219 (86.2%) reported that they intend to continue using the JSANDS system to register neonatal deaths and stillbirths in the future, and 213 (83.9%) reported that they intend to use it even without having any incentives.

#### Penetration

Of the 270 participants, 14 (5.5%) users used it once, 70 (27.6%) used it but not frequently, 81 (31.9%) used it at least once a weak, and 89 (35.0%) used it every day.

#### Data Quality and Sensitivity

In Jordan, all births and deaths should be registered and reported to the Civil Status and Passports Department (CSPD). To investigate data quality and sensitivity of the JSANDS, we compared registered deaths on the system with deaths reported to the CSPD for the same period. Further, all data entered on the system were carefully checked by comparing them with data in maternal or child medical records. We found that all variables had complete data with no missing values. Unsurprisingly, all neonatal deaths reported to the CSPD (that used paper notification) from the selected hospitals during the same pilot period were registered in JSANDS. On the other hand, 85.0% (648/762) of neonatal deaths and all stillbirths registered by JSANDS were not registered in the CSPD, neither as births nor as deaths.

## Discussion

Counting deaths, collecting information about causes of these deaths, and determining the contributing and avoidable factors are essential for delaying unnecessary early death events and improving the health care system [10]. Meanwhile, designing electronic registration and reporting systems for births and neonatal deaths is essential for reducing stillbirths and neonatal mortality [11].

This study showed that the users of the JSANDS found the system to be useful, functional, and high-performing. In a previous study on the usability of JSANDS, health professionals rated it positively as indicated by the high usability ratings, satisfaction with the system, and ratings of the helpfulness of the system in making important decisions with quality of care [12].

JSANDS was rated by the system users as useful because it provides estimates of the PNN mortality rate and the causes of deaths overall according to other social and clinical determinants. This finding is supported by the fact that the JSANDS data were used by authors in previous studies [13,14] to determine the rate, determinants, and causes of stillbirths and neonatal deaths in Jordan. The researchers in these studies reported that the majority of neonatal deaths could have been prevented and have recommended specialized care to be provided to low–birth-weight neonates and those with respiratory problems by experienced health care providers [13]. Moreover, the researchers reported that the majority of stillbirths registered by JSANDS occurred during the antepartum period, and they recommended that care should be taken for the early identification of high-risk pregnancies and ensuring adequate antenatal obstetric interventions [14].

The system also reports data on other maternal and child health indicators, such as cesarean section rate, preterm delivery rate, and low–birth-weight delivery rate. For example, the system showed that the cesarean section rate is extremely high, reaching a rate of almost 50%. The finding of the high cesarean section rate in Jordan is consistent with the findings of previous studies [15,16].

Reporting mortality estimates by social determinants is useful for informing health care decision making and program evaluation to support the equitable delivery of essential and quality interventions to populations in need. JSANDS collects the data that are necessary to create equity indicators. Combining mortality measures and equity stratifiers in particular ways can yield policy-relevant information that also reveals the basic inequities in society. Having a range of health measures and equity stratifiers facilitates timely recognition of emerging or hidden inequities and improves accountability for protecting vulnerable populations. Currently, the JSANDS dashboard disaggregates mortality measures by gender, nationality, geographic location, and socioeconomic status to clarify if there are any significant differences present.

During the implementation of JSANDS, the availability of data on deaths and their causes triggered health professionals in the hospitals to intervene and improve the quality of services. Several success stories were documented, demonstrating the attempts of health professionals to improve the quality of maternal and neonatal services [17].

JSANDS users rated acceptability and all its indicators as excellent except 1 indicator, “Using JSANDS is time-consuming.” One reason for perceiving its use as time-consuming is that JSANDS collects additional information on social determinants of deaths. Including these data is justified as it can improve health equity and reduce disparities in care.

Regarding stability, 3 indicators were rated excellent and 2 indicators were rated good. Although users thought that the stability would be affected by the withdrawal of sponsor support, the JSANDS managers believe that the system will maintain its stability after the end of sponsors’ funding because the system is not resource demanding. All hospitals have a sufficient number of computers, and any one of these computers can be used to install JSANDS. The 12 computers that were used for running JSANDS in the pilot hospitals were functional and did not need repair during the period of implementation. The number of unscheduled outages and down times for the system's computers during the 18 months of implementation was 8 times over all hospitals, and this did not affect the data registration or reporting. The system was operating fully at all times.

The simplicity of JSANDS was rated as good. Five of the simplicity indicators were rated good. It seems that the focal points need continuous and frequent trainings to use the system. To ensure that the focal points are aware of the system, an online manual on JSANDs and its use is available at the JSANDS website [17]. From our own experience in managing JSANDS and training, only 2 hours of training are needed to train people on using the system. Moreover, JSANDS users thought that JSANDS requires too much information to register neonatal death and stillbirths. Looking at an objective indicator of time needed to enter the data into JSANDS, the time spent on collecting and entering the data for registering a birth ranged from 3 to 5 minutes while the time needed for registering a neonatal death and stillbirth was 2 and 3 minutes, respectively. Once data about each case are entered into the system at the health facility, the data are transferred immediately to the Ministry of Health. Moreover, the system automatically analyzes the data and produces the indicators and reports promptly once they are requested.

JSANDS was piloted in 5 hospitals, and the representativeness of deaths cannot be assessed. However, the system was able to accurately describe the occurrence of PNN deaths over time and its distribution in the selected hospitals. On the other hand, users reported that the system can be used in all health sectors. With the use of more objective measures of representativeness, all births, stillbirths, and neonatal deaths that occurred in the selected hospitals were registered and reported by JSANDS.

In conclusion, JSANDS was shown to be a reliable and useful system for registering and reporting stillbirths and neonatal deaths and their causes in Jordan. This real-time surveillance system enables health care professionals to detect unusual increase in stillbirths and neonatal deaths and to detect the exact root of the noticeable deaths at a particular time. The performance of JSANDS in fully registering stillbirths and neonatal deaths and their causes was excellent. Almost all attributes and indicators of JSANDS functionality were rated excellent. JSANDS can be scaled up to cover all maternity hospitals in Jordan. The potential for scaling up the system is very high for many reasons, including its usefulness, the simplified PNN death review tools, and its easy-to-use reporting process.

## Abbreviations

CDC: Centers for Disease Control and Prevention
CSPD: Civil Status and Passports Department
ICD-10: International Classification of Diseases Tenth Revision
JSANDS: Jordan Stillbirth and Neonatal Mortality Surveillance
PNN: perinatal and neonatal
UNICEF: The United Nations Children's Fund
